# Supplementary material for: The Impact of SARS-CoV-2 Primary Vaccination in a Cohort of Patients Hospitalized for Acute COVID-19 during Delta Variant Predominance
Source: J Clin Med. 2022 Feb 23;11(5):1191. doi: 10.3390/jcm11051191 (PMC8911274; doi:10.3390/jcm11051191)

Supplementary Table S1. Categorization of underlying chronic illnesses.

Chronic Lung Disease	• Asthma<br>• Cystic fibrosis<br>• Chronic obstructive pulmonary disease<br>• Obstructive sleep apnea<br>• Oxygen dependence<br>• Pulmonary fibrosis<br>• Restrictive lung disease<br>• Sarcoidosis
Chronic Metabolic Disease	• Adrenal Disorders<br>• Diabetes mellitus<br>• Glycogen or other storage diseases<br>• Hyper-/Hypo-pituitary function<br>• Hyper/Hypoparathyroidism<br>• Congenital metabolic diseases<br>• Metabolic Syndrome<br>• Thyroid dysfunction
Hematologic Conditions	• Chronic anemia<br>• Coagulopathy or other bleeding disorder<br>• Myelodysplastic syndrome<br>• Polycythemia vera<br>• Sickle cell disease<br>• Thalassemia<br>• Thrombocytopenia
Cardiovascular Disease	• Atherosclerotic cardiovascular disease<br>• Cardiac arrhythmias<br>• Cardiomyopathy<br>• Congenital heart disease<br>• History of aortic aneurysm<br>• History of transient ischemic attack/Stroke<br>• Heart failure<br>• Heart valve disease<br>• Hypertension<br>• Peripheral artery disease<br>• Pulmonary hypertension
Neurologic Disorders	• Amyotrophic lateral sclerosis<br>• Cerebral palsy<br>• Cognitive dysfunction<br>• Dementia/Alzheimer's disease<br>• Developmental delay<br>• Epilepsy<br>• Multiple sclerosis<br>• Muscular dystrophy<br>• Myasthenia gravis<br>• Parkinson's disease<br>• History of brain injury<br>• History of Guillain-Barre syndrome<br>• Paraplegia/Quadriplegia
Immunocompromising Conditions	• Active treatment with immunosuppressive medication<br>• Complement deficiency<br>• Hematologic cancer [1]<br>• History of hematopoietic stem cell transplant within 2 years<br>• History of solid organ transplant<br>• History of splenectomy<br>• HIV infection<br>• Immunoglobulin deficiency

	• Solid organ malignancy [1]
Renal Disease	• Chronic kidney disease<br>• Dialysis<br>• End stage renal disease<br>• Glomerulonephritis<br>• Nephrotic syndrome<br>• Polycystic kidney disease
Gastrointestinal/Liver Disease	• Alcoholic hepatitis<br>• Autoimmune hepatitis<br>• Barrett's esophagitis<br>• Chronic liver disease<br>• Chronic pancreatitis<br>• Cirrhosis/End stage liver disease<br>• Crohn's disease<br>• Esophageal varices<br>• Hepatitis B, chronic<br>• Hepatitis C, chronic<br>• Non-alcoholic fatty liver disease<br>• Ulcerative colitis
Rheumatologic/Autoimmune Conditions	• Ankylosing spondylitis<br>• Dermatomyositis<br>• Juvenile idiopathic arthritis<br>• Microscopic polyangiitis<br>• Polyarteritis nodosum<br>• Polymyalgia rheumatica<br>• Polymyositis<br>• Psoriatic arthritis<br>• Rheumatoid arthritis<br>• Systemic Lupus Erythematosus<br>• Temporal/Giant Cell arteritis<br>• Vasculitis, other
Other	• Other underlying medical conditions not specified above

[1] Currently treated or diagnosed in the past 6 months

Supplementary Table S2. Association between prior vaccination and progression to critically severe disease including two patients with unresolved status assumed to have progressed to critically severe disease (the World Health Organization COVID-19 Clinical Progression Scale 7–10) [12] and subdistribution hazard ratios for progression to critically severe disease.

**Characteristics**	**Odds ratio (95% CI)**	***P* value**	**Subdistribution hazard ratio (95% CI)**	***P* value [1]**
Intercept	0.11 (0.03–0.37)	**<0.001**		
Vaccination status (vaccinated vs. unvaccinated)	0.41 (0.24–0.67)	**<0.001**	0.48 (0.32–0.73)	**<0.001**
Age	1.01 (0.99–1.03)	0.403	1.01 (0.99–1.03)	0.410
Sex (male vs. female)	1.40 (0.91–2.15)	0.124	1.42 (0.98–2.03)	0.061
Charlson comorbidity index	1.17 (1.01–1.36)	**0.039**	1.12 (1.02–1.25)	**0.024**
Immunocompromising condition present (yes vs. no)	1.03 (0.45–2.38)	0.941	1.08 (0.57–2.03)	0.820

CI, confidence interval

[1] *P* value < 0.05 was considered significant (marked in bold).

Supplementary Table S3. Cause-specific hazard ratios for discharge from hospital.

**Characteristics**	**Hazard ratio (95% CI)**	***P* value [1]**
Vaccination status (vaccinated vs. unvaccinated)	1.37 (1.09–1.70)	**0.006**
Age	1.00 (0.99–1.01)	0.485
Sex (male vs. female)	0.90 (0.74–1.09)	0.271
Charlson comorbidity index	0.94 (0.86–1.01)	0.107
Immunocompromising condition present (yes vs. no)	0.91 (0.61–1.35)	0.629

CI, confidence interval

[1] *P* value < 0.05 was considered significant (marked in bold).

Supplementary Figure S1. Number of patients with PCR-confirmed vaccine breakthrough SARS-CoV-2 infections admitted in relation to time between last vaccine dose and hospital admission in weeks.

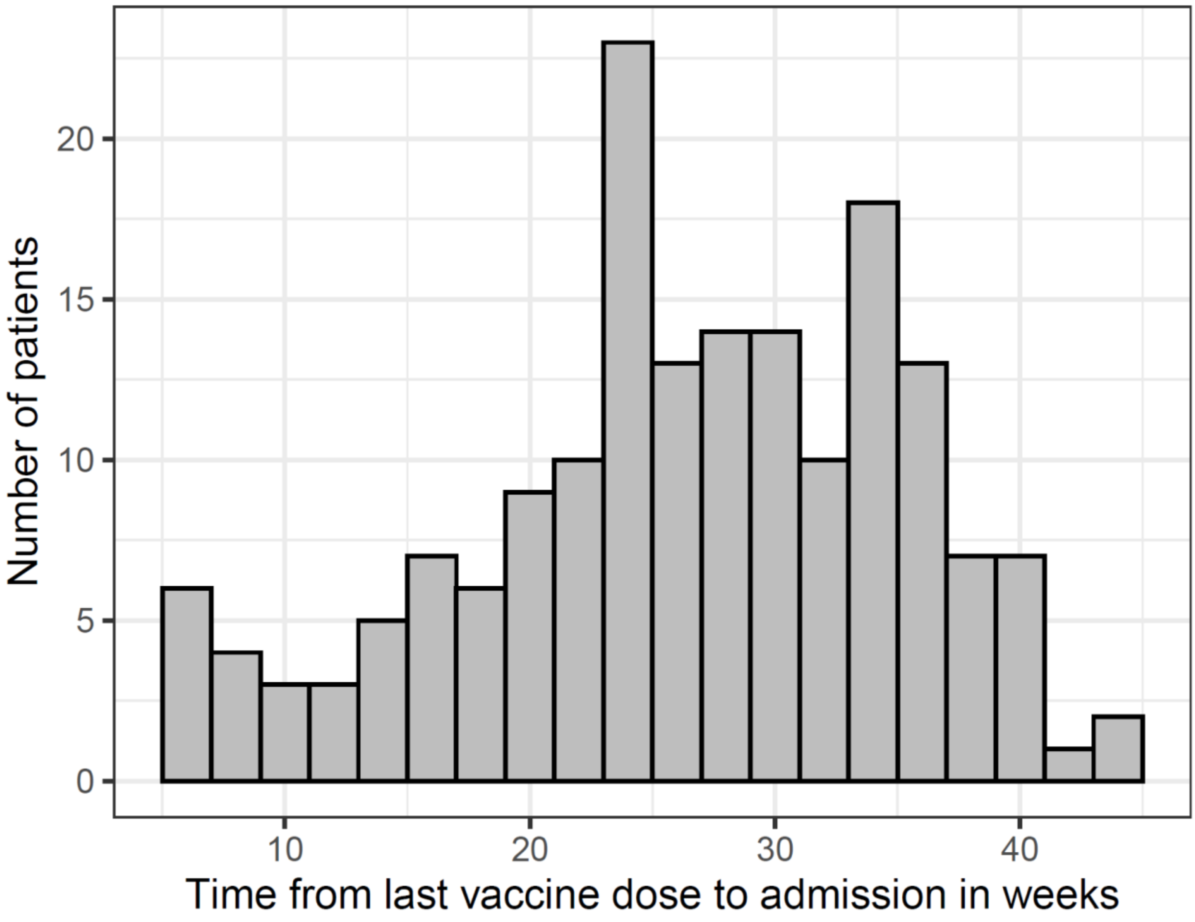

Supplement: Supplementary file 1 [file jcm-11-01191-s001.zip › jcm-1607265-supplementary.pdf]
